# Developmental Screening Tools: Feasibility of Use at Primary Healthcare Level in Low- and Middle-income Settings

**Published:** 2014-06

**Authors:** Vinicius Jobim Fischer, Jodi Morris, José Martines

**Affiliations:** ^1^Faculty of Psychology, Pontifical University Catholic of Rio Grande do Sul and School of Physical Education, Federal University of Rio Grande do Sul, Brazil; ^2^Department of Mental Health and Substance Abuse, World Health Organization, Geneva, Switzerland; ^3^Department of Maternal, Newborn, Child and Adolescent Health, World Health Organization, Geneva, Switzerland

**Keywords:** Child development, Developmental disabilities, Feasibility, LMIC, Primary healthcare, Screening tools

## Abstract

An estimated 150 million children have a disability. Early identification of developmental disabilities is a high priority for the World Health Organization to allow action to reduce impairments through Gap Action Program on mental health. The study identified the feasibility of using the developmental screening and monitoring tools for children aged 0-3 year(s) by non-specialist primary healthcare providers in low-resource settings. A systematic review of the literature was conducted to identify the tools, assess their psychometric properties, and feasibility of use in low- and middle-income countries (LMICs). Key indicators to examine feasibility in LMICs were derived from a consultation with 23 international experts. We identified 426 studies from which 14 tools used in LMICs were extracted for further examination. Three tools reported adequate psychometric properties and met most of the feasibility criteria. Three tools appear promising for use in identifying and monitoring young children with disabilities at primary healthcare level in LMICs. Further research and development are needed to optimize these tools.

## INTRODUCTION

An estimated 150 million children suffer from some kind of disability (1), and over 200 million are not fulfilling their developmental potential (2). Prevalence data are scarce on children below 3 years due to the limited availability of tools to assess young children and the lack of simple yet reliable and valid instruments that can be used in large surveys. Most of these children live in the poorest parts of the world (1). These children often do poorly in school, are less likely to be productive adults, and are at increased risk of transferring poverty to the next generation (2,3). The World Health Organization has made early identification of children below 3 years with disabilities a high priority, especially as identification at this young age may reduce the impact of impairments (4). Interventions to promote development of young children are known (5) and are increasingly becoming available in low- and middle-income countries (LMICs). These include addressing malnutrition and iron deficiency, improving caregiver-child relationship and psychosocial stimulation, and establishing community-based rehabilitation (6).

The WHO has recently launched an evidence-based clinical guideline for assessment and management of priority mental, neurological and substance-abuse conditions by non-specialist primary care workers through Mental Health Gap Action Program (mhGAP) Intervention Guide (7). Because of the high disability burden and the associated financial costs and human rights violation associated with developmental disorders, these are among the conditions addressed by the mhGAP Intervention Guide. The guide provides decision-making flowcharts for detection and management of developmental disorders at primary healthcare level. However, the lack of tools for assessment and monitoring of child development, suitable for use by non-specialists in low-resource settings, hampers the possibility of mainstreaming mhGAP in child healthcare services.

Identification of infants who are in need of early intervention requires the use of a valid developmental diagnostic assessment tool (8). While standardized tools from western countries provide assessment tests that have been well-validated in their settings, the transfer of western-based tests to non-western contexts is associated with significant limitations of score interpretation and feasibility of use in resource-constrained settings (9,10).

An important challenge in early identification of developmental disability is having tools that respond to local differences, including cultural perceptions in meaning of disability and can be used across countries.

When comparing test responses across populations that differ in language and other aspects of culture, the comparability of the assessment procedures is a special concern. Cross-cultural equivalence is especially problematic when assessments depend on verbal reports of individuals sampled from the population. In such instances, it is essential to show that population characteristics, such as preferred language, level of education, and cultural values, do not affect the quality of the assessment (11).

Although tools can play an important role in identifying children who can benifit from interventions, the wisdom of applying these in settings where specialized training is not widely available and contacts with the health services are constrained is highly questionable. Efforts to identify children with disabilities are only justified when they can lead to interventions. In the context of the increased opportunities of access to care created by mhGAP, the present study was conducted to review the available literature to identify developmental monitoring and screening tools that have been used in LMICs for children aged 0-3 year(s) and to evaluate these tools by examining their psychometric properties (sensitivity, specificity, validity, reliability), the requirements for their application, and the feasibility of their use in LMICs.

The focus on this group has two main reasons: (i) children below the age of 3 years have more frequent contacts with health facilities, an important opportunity for identification and management of disabilities and (ii) children at such young age are more responsive to interventions.

## MATERIALS AND METHODS

The study was conducted in two parts. In the first part, we searched for literature reviews that were completed since 2007 examining tools for identification of children with developmental disabilities. The search included articles published since 2007 in order to ensure that we would identify any possible articles which, due to late publication or indexing, might not have been captured by existing reviews (12-14).

Three reviews were identified (12-14), the latest dating from 2010 (14). To supplement and update the latest review, a systematic search was conducted on Eric, PubMed, Psycinfo, and Scielo, using an adaptation of the search used in the review (14) but restricting the search to the age-group of interest (children up to 3 years). We applied the search terms (Annex) looking for publications since 2009.

Identified papers were selected for possible inclusion based on the following criteria: (a) studies conducted with tools to assess multiple child development aspects (tools regarding more than one of the developmental domains: motor, cognitive, language, socio-emotional); (b) tools applied to children aged 0 to 3 year(s); (c) studies conducted in LMIC; and (d) studies that presented information on the tool's performance.

We identified 426 articles. After applying the selection criteria, 20 were retained. Details of the search are presented in the figure that presents the sources of information used in the study. Most of the studies included were identified from the 3 pre-existing reviews. Six additional studies were identified through PubMed; searches in Eric, Psycinfo, and Scielo did not add further studies.

We extracted information on the characteristics of the tool (age range of applicability, training needed to apply the tool, time and costs of its use, specificity, sensitivity, positive predictive value, negative predictive value, other validity and reliability properties) and of the study that evaluated it (sample-size and age-range, professional background of persons who administered the tool, and whether the used tool was culturally adapted for application in the study).

The second part of our study consisted of deciding what aspects to examine when assessing the feasibility of applying the tool in healthcare settings in LMICs. For this, we consulted a group of international experts. We started the consultation by generating a set of tool characteristics that we believed would indicate the degree of the tool application being easily or conveniently done (according to the definition of ‘feasibility’ in the Dictionary of Military and Associated Terms. US Department of Defense, 2005) (15). This list of characteristics was sent to 23 experts on child development, who were asked to score each of these from 1 to 5 according to their perception of the importance of the characteristics in determining the feasibility of applying the tool. They were also asked to add characteristics that they felt would be important but had not been included in our original list. After compiling the scores, we selected the 9 characteristics most valued by the experts. Using information from the papers identified in Part 1, this set of characteristics was then used in identifying which tools were likely to be the most feasible for implementation in LMICs. The criteria were developed by consensus among a group of WHO experts on the implementation of mental health and child health programmes.

**Figure.**
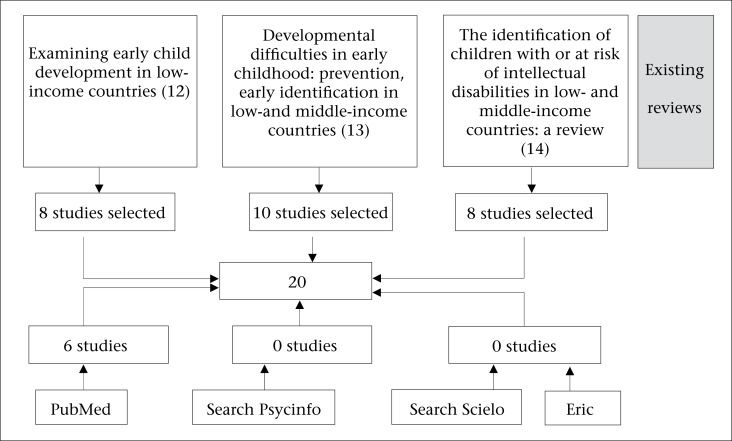
Information sources

## RESULTS

Twenty tools were identified as available for screening developmental disabilities. Some of these were developed in high-income countries while most were designed for low- and middle-income settings.

### Tools and their properties

#### Tools developed in high-income countries

We identified six internationally-used tools to assess cognition and other domains in infants from 0 to 3 year(s) of age. Bayley Scales for Infant Development-II (BSID-II), British Ability Scales, Denver Developmental Materials, Stanford-Binet, Ages and Stages Questionnaire, Vineland Adaptative Behavior Scales II (Box 1). We looked for studies that aimed to validate these tools in LMICs. However, most of the studies in LMICs used these tools as the gold standard and compared the locally-developed tools with one or more of these. This comparison often happened, despite the lack of adaptation of the international tools for countries of use (16,17). Examples of such comparisons are presented below.

BSID-II: Differences in cross-cultural performance have been described when comparing infants from England, Mexico, Brazil, and Taiwan (8,18).

Denver Test: Drachler *et al*. (19) compared the results of Brazilian infants between 6 and 59 months of age in the Denver Test with the response given by mothers to the request to compare their children's actions with other children of similar age and to report whether the children were advanced, delayed, or comparable. Results indicated high specificity (over 91%) but variable sensitivity ranging from 33% among mothers of four or less years of education to 73% among mothers of nine or more years of education. Similar variability was observed when comparing the responses of mothers according to income group.

**Box 1. Tools developed in western countries**

**Table:** 

Bayley Scales of Infant Development (BSID-I, 1st edition; BSID-II, 2nd edition; BSID-III, 3rd edition)	Assess the developmental status of infants and children in a wide range of domains. The primary value of the test is in diagnosing developmental delay and planning intervention strategies
British Ability Scales (BAS)	Measures core (verbal, visual/spatial, and non-verbal) as well as subscales for differential abilities and, achievement tests in the older group. Purpose is the assessment of particular cognitive abilities linked to developing understanding and supporting interventions rather than categorization of children. This facilitates the movement away from the restrictive practice of generating broad and general assessment information across a range of cognitive abilities with a focus on categorization rather than intervention
Denver Developmental Materials II (formerly DDST)	This is a surveillance and monitoring tool used by professionals or trained paraprofessionals to determine if a child's development is within the normal range. The results are not diagnostic. The DENVER II is designed to reflect the development of a broad range of heterogeneous skills in a minimum amount of time. As such, it is not designed to measure any single construct, such as intelligence, motor functioning, or social skill.
Stanford Binet Intelligence Scale	This test is used for studying the development of cognitive skills of individuals. The measure contains 15 subtests that assess mental abilities in four areas: verbal reasoning, abstract visual reasoning, quantitative comprehension, and short-term memory
Ages and Stages Questionnaire (ASQ)	To screen infants and young children for developmental delays during the first 5 years of life. The assessment covers five key developmental areas: communication, gross motor, fine motor, problem solving, and personal-social skill
Vineland Adaptative Behavior Scales II	Used in identifying individuals who have intellectual and developmental disabilities from birth to 90 years of age. It includes 4 domains: communication, daily living skills, socialization and motor skills

Denver II, DDST and Griffith's tools: Gladstone *et al*. (20) applied this set of western tools in a rural population of Malawi to assess their performance as screening tools. While gross motor items were mostly retained for their good performance (97%), just over 50% of the social items performed well enough to be retained. She concluded that, although many items of Denver II, DDST, and Griffith's tools can be adapted, the gross motor domain is more culturally adaptable than the social developmental domain.

DDST and Denver II: These tools were adapted by Lim *et al*. (21) to the Singapore population. In order to make the tools suitable, these had to be adapted taking into account the ethnicity and mother's education variability that affected social habits, child's language, and gross motor development.

#### Tools developed in LMICs

Fourteen tools developed in LMICs were identified: Baroda Development Screening Test (BDST), Developmental Assessment Tool for *Anganwadis* (DATA), Disability Screening Schedule (DSS), Ten Questions Screen (TQS) for Childhood Disability, Kilifi Developmental Inventory (KDI), Trivandrum Developmental Screening Chart (TDSC), Guide for Monitoring Child Development (GMCD), Screening Test Battery for Assessment of Psychosocial Development (STBAPD), Parents Evaluation of Developmental Status (PEDS), Comprehensive Developmental Inventory for infants and Toddlers (CDIIT), Rapid Neurodevelopmental Assessment Tool (RNDA), Malawian Developmental Assessment Tool (MDAT), Lucknow Developmental Screen, and Angkor Hospital for Children Developmental Milestone Assessment Tool (AHC DMAT) to assess child development from age 0 to 3 year(s). Box 2 provides a description of these tools. Information on their performance is summarized here.

The Baroda Development Screening Test (BDST) was developed by Phatak and Khurana (22) to assess motor and mental development of infants in Baroda, India. The tool can be used for children aged 0 to 30 month(s). It is based on the Bayley Scales of Infant Development and is composed of 54 items (22 focus on motor and 32 on mental development). Selected items were those found to be simple to administer not requiring special training, experience, and equipment. The BDST is described as a simple and quick tool that can be applied by health workers in a door-to-door survey. Its specificity was 65% and sensitivity 95% when tested in Pune, India, comparing the assessment by health workers with that by fully-trained testers.

Developmental Assessment Tool for *Anganwadis* (DATA) developed by Nair *et al*. (23) is a tool that is simple to apply, short, not expensive, and requires limited training. It has been reported to be able to identify children who have developmental delays at 2.5 years. No information regarding specificity and sensitivity was found.

The Disability Screening Schedule (DSS) was developed by Chopra *et al.* (24) to be a one-time screen for major disabilities (physical, motor, sensory and mental retardation). The DSS seeks information on prenatal and birth history, physical and sensory functions, and developmental assessment of the child. Screening information is obtained through parental reports, observations of the child, and assessment of task performance by the child. It can be applied to children between 0 and 6 year(s) and is considered easy to use, readily comprehensible, and quick to administer (around 5 minutes). The sensitivity was 0.89 and specificity 0.98 in identifying disabilities. A positive predictive value of 0.89 and a negative predictive value of 0.98 were estimated in the test population.

The Ten Questions Screen (TQS) for Childhood Disability was developed to serve as a rapid, low-cost tool to assist in the identification of children with serious disabilities in population of limited resources. Durkin *et al.* (11) evaluated the tool among 2 to 9 years old children when applied by trained community health workers in Bangladesh, Jamaica, and Pakistan. The specificity of the TQS for severe disability was high in all three populations and constant across types of disabilities (0.92 in Bangladesh, 0.85 in Jamaica, and 0.86 in Pakistan). Sensitivity for cognitive disability was 0.82 in Bangladesh and 0.84 in Pakistan but only 0.53 in Jamaica. Nonetheless, when considering severe cognitive disability, it was 1.00 in all three populations. The sensitivity of the tool was low for mild disabilities. When assessed by professionals, severe non-sensory disabilities were identified ranging from 80 to 100% of the cases. The tool has been found to have relatively poor sensibility for serious visual and hearing disorders (18).

The Kilifi Developmental Inventory was designed to assess psychomotor functioning of children aged 6 to 35 months. It consists of 69 activity items explained and demonstrated before the child tries the activity. The items were selected from various sources, including the Griffiths Mental Developmental Scales, Wessex Revised Portage Checklist, the Wechsler Preschool and Primary Scales of Intelligence, and the Kenyan Screening Test for children aged 6 months to 6 years (10). Its evaluation in Kenya indicated high community acceptability, internal consistency (over 90%), inter-observer agreement (over 90%), and retest reliability (over 85%). Results show a significant association between performance, age, and anthropometric status. No information regarding specificity and sensitivity was found.

The Trivandrum Developmental Screening Chart (TDSC) by Nair *et al.* (23) was designed to assess mental and motor development over the first two years of life. It is a simple tool, it can be applied by health workers over 5 minutes and only requires a pen and a bunch of keys as test items. It consists of 17 test items with a concentration of items near 1 year of age. Its validation against Denver Developmental Screening Test indicated the TDSC to have a relatively low sensitivity of 66.7% and specificity of 78.8% in identifying developmental delays.

The Guide for Monitoring Child Development (GMCD) by Ertem *et al.* (6) is a tool designed for developmental monitoring and early detection of children's developmental difficulties in LMICs. It consists of a brief, open-ended, pre-coded interview with the primary caregiver that can be applied for children from 0 to 2 year(s) of age. The first question relates to identifying parents’ concern. Question 2 to 7 relate to: expressive language and communication, receptive language, gross and fine motor, relationship (socio-emotional), play and self-help skills (for children older than 12 months). The tool has been tested in a population with a high proportion of mothers with less than 5 years of schooling. The tool was applied by medical students who found it easy to apply and to communicate with caregivers. The mean±SD administration time was 7±2.3 minutes. Mothers found the questions easy to understand and answer. The inter-rater reliability comparing health workers (medical students) with child development specialists ranged between 92.3% and 94.5%. Agreement tended to be higher when applied to mothers with more years of education. The sensitivity of the tool was 0.88 (95% CI 0.69-0.96). The specificity was 0.93 (95% CI 0.83-0.97). The positive predictive value was 0.84, and the negative predictive value was 0.94.

**Box 2. Description of tools developed in LMICs**

**Table:** 

Tool	Purpose	Domains measured	Time taken	Sensitivity and specificity
Baroda Development Screening Test	Assess motor and mental development of infants	Motor and mental	Quick	Sensitivity 95% Specificity 65%
Developmental Assessment Tool for *Anganwadis* (DATA)	Identify children with developmental delays	Gross motor; fine motor; cognitive; personal; social; expressive language; receptive language	Short	Not available
Disability Screening Test (DSS)	To screen major disabilities	Physical, motor, sensory and mental retardation	Around 5 minutes	Sensitivity 89% Specificity 98%
Ten Questions Screen for Childhood Disability	Identify children with serious disabilities in population of limited resources	Cognitive disability; movement disability; seizures; vision; and hearing	Brief	Sensitivity: For cognitive disability. 82% in Bangladesh; 84% in Pakistan; and 53% in Jamaica; For severe cognitive disability, 100%; For non-sensory disabilities, 80 to 100% Specificity: For severe disability, 92% in Bangladesh; 86% in Pakistan; and 85% in Jamaica
Kilifi Developemental Inventory	Assess psychomotor functioning	Eye-hand coordination and locomotor skills	Not available	Not available
Trivandrum Screening Chart (TDSC)	Assess mental and motor development	Mental; motor development; hearing and visual functions	Around 5 minutes	Sensitivity 66.7% Specificity 78.8%
Guide for Monitoring Child Development (GMCD)	Assess developmental monitoring and early detection of developmental difficulties	Communication; gross and fine motor; socio-emotional; play self-help skills	7±2.3 minutes	Sensitivity 88% Specificity 93%
Screening Test Battery for Assessment of Psychosocial Development	Screen developmental delays	Gross motor vision and fine motor; hearing; language and concept development; self-help skills; and social skills	Not available	Not available
Parents’ Evaluation of Developmental Status (PEDS)	Assess parents’ concerns on child's learning, development and behaviour	Learning; development and behaviour	Short	Sensitivity 61.5% Specificity 65.1%
Comprehensive Developmental Inventory for Infants and Toddlers (CDIIT)	Assess child development	Cognitive; language; motor; social and self-help skills	45 to 90 minutes	Not avaliable
Rapid Neurodevelopmental Assessment Tool	Determine functional status	Primitive reflexes; gross motor; fine motor; vision; hearing; speech; cognition; behaviour and seizures skills	45 minutes, on average	Not available
Malawian Developmental Assessment Tool (MDAT)	Create a culturally-appropriate developmental assessment tool for rural Africa	Gross motor; fine motor; language and social skills	Around 30 minutes	Sensitivity 97% Specificity 82%
Lucknow Development Screen	Create a valid and reliable screening tool for children from 6 months to 2 years of age	Gross motor; fine motor; language and social skills	10 minutes	Sensitivity 95.9% Specificity 73.1%
Angkor Hospital for Children Developmental Milestone Assessment Tool	Create a culturally-appropriate neurodevelopmental screening tool	Gross motor; fine motor; language and social-personal aspects skills	15 to 20 minutes	Not available

The Screening Test Battery for Assessment of Psychosocial Development (STBAPD) was developed by Vazir *et al.* (25) to screen developmental delays in children aged 0 to 6 year(s) in rural India. The battery is composed of 66 items divided in 5 areas: (i) gross motor, (ii) vision and fine motor, (iii) hearing language and concept development, (iv) self-help skills, and (v) social skills. The tool was found to be culturally appropriate, simple and easy to be administered by community health workers with seven or more years of education. Training to apply the battery included two theory classes on principles of child development and method of assessment and two weeks of practical training consisting of demonstration on how to administer and score the items. Inter-tester reliability was calculated between the supervising psychologists and the community health workers; coefficients ranged from 95 to 98%. The retest reliability coefficients ranged from 95 to 99%. No information regarding specificity and sensitivity was found.

The Parents’ Evaluation of Developmental Status (PEDS) is a tool that can be applied to young children. It includes an open-ended question to parents asking about any concerns on the child's learning, development, and behaviours Additional questions probe developmental concerns in each domain. It is short and easy to administer. An assessment of the performance of the PEDS was conducted in Chandigarh, India, with children aged 2 to 5 years by Malhi and Singhi (26). The presence of significant parental concern identified 61.5% of children with delayed development (sensitivity). Of children with normal development, 65.1% of the parents were accurately non-concerned (specificity). The positive predictive value was 25.8%, and the negative predictive value was 89.6%. There were no differences between the accurately concerned and inaccurately non-concerned parents in sociodemographic variables, such as level of education and income. The sensitivity and specificity of PEDS in this study was lower than that reported for North American children aged 0 to 7 year(s) (sensitivity 75% and specificity 74%).

The Comprehensive Developmental Inventory for Infants and Toddlers (CDIIT) was developed in Taiwan by Liao *et al.* (27). It consists of subtests of cognitive, language, motor, social and self-help subtests, and a ranking behaviour record. Its application requires training by a specialist and application takes around 45 to 90 minutes. The CDIIT was validated against the Bayley Scales of Infant Development-II for Taiwanese population for children aged 3 to 71 months. For developmental ages, the motor and mental scales of CDIIT were highly correlated with the BSID-II but less so for developmental quotients. The CDIIT Developmental Quotients tended to classify children at higher developmental levels than the BSID-II. No information regarding specificity and sensitivity was found.

The Rapid Neurodevelopmental Assessment Tool (RNDA) (28) was designed in Bangladesh to determine functional status in the following domains: primitive reflexes, gross motor, fine motor, vision, hearing, speech, cognition, behaviour, and seizures of children aged 0 to 2 year(s). RNDA was administered by professionals with a minimum work experience of 4 years. The administration and scoring took, on average, 45 minutes for children <1 month and 30 minutes for children aged 1 to 24 month(s). Reliability and validity were determined for children below 3 months and from 3 to 24 months, with a simultaneous administration of the adapted version of the Bayley Scales of Infant Development (BSID) by a psychologist as the gold standard. For children aged <3 months, reliability was high in 6 domains (with kappa ranging between 0.83 and 1) and good in 3 domains (with kappa ranging between 0.76 and 0.78). For children aged 3 to 24 months, reliability was high in 5 domains (with kappa ranging between 0.85 and 1) and was good in 3 domains (with kappa ranging between 0.63 and 0.80). Concurrent validity was good between the RNDA and the BSID for children aged <3 months but inferior for children from 3 to 24 months of age. No information regarding specificity and sensitivity was found.

The Malawian Developmental Assessment Tool (MDAT) (29) was developed by Gladstone *et al.* aiming to create a culturally-appropriate developmental assessment tool for use in rural Africa. The tool can be applied to children aged 0 to six year(s). It is composed of 136 items divided in four domains: gross motor, fine motor, language, and social domain, with 34 items each. The MDAT takes approximately 30 minutes to administer and can be used by local health workers with little training. Reliability of assessments (k>0.75) was found to be 99% (immediate), 89% (delayed), and 71% (2-week delayed). Sensitivity was found to be 97% and specificity 82%.

The Lucknow Development Screen for Indian Children was designed by Bhave *et al.* in India to create a valid and reliable screening tool for children from 6 months to two years of age (30). It is composed of 27 milestones, including gross motor, fine motor, language and social domains which cover each month of age and beyond. The tool can be administered to the caretaker as a verbal structured interview and takes 10 minutes to administer. The Lucknow Development Screen was used in screening 142 children and was validated against the Developmental Assessment Scale for Indian Infants, and, in 3 cases, against the Vineland Social Maturity Scale. Sensitivity was found to be 95.9% and specificity 73.1%.

The Angkor Hospital for Children Developmental Milestone Assessment Tool (AHC DMAT) was developed in Cambodia by Ngoun *et al.* to create a culturally-appropriate neurodevelopmental screening tool for assessing Cambodian children (31). The AHC DMAT consists of 140 milestones (49% derived directly from DDST II, 17% modified from DDST II, 34% added through expert opinion) and can be applied to children from 1 month to 6 years of age. The milestones are divided in four domains: gross motor, fine motor, language, and social-personal aspects. Evaluation was conducted by screeners/investigators based on child demonstrations but parent's/caretaker's report was also allowed. The average time to administer the tool ranged from 15 to 20 minutes. No information regarding specificity and sensitivity was found.

Out of the 14 tools developed in LMICs that we identified, we were able to obtain information on the sensitivity and specificity for 8. Out of these, only 4 showed sensitivity and specificity greater than 80%. These tools are: DSS, Ten Questions, GMCD, and MDAT.

### Feasibility criteria

Eighteen of the 23 experts who were initially contacted provided scores for feasibility characteristics. Based on their indication, five additional respondents were contacted. The total response rate was 82.14% (23 out of 28). The responses are summarized in Table 1. The eight characteristics that received the highest scores were selected to examine when to consider implementation feasibility at PHC of tools to detect developmental disabilities. The characteristics selected were: costs of the tool, access to application, training required, time to administer the tool, validity, reliability, results useful to guide action, and results understood by caregivers and workers.

### Assessment of the feasibility of applying the tools in LMICs

Based on the available data, each criterion was given a score of 1 (fulfilled) or 0 (not fulfilled). Missing information was noted.

We stipulated that costs of the tool should be nil, access to the tool should be easy and downloadable from the Internet. Training required to use the tool should take no longer than 3 days; time to administer the tool should take no longer than 30 minutes. Regarding validity and reliability, the values should be acceptable for developmental screening tools (32). Results should be understood by caregivers and PHC workers and guide further action.

Out of 112 feasibility characteristics considered (8 feasibility characteristics for 14 tools), information was missing on 60. Only for 3 tools (Ten Questions Questionnaire, GMCD, and MDAT), we identified information on 4 or more of the feasibility criteria. The information on the performance of these tools is presented in Table 2.

## DISCUSSION

Out of the extensive search conducted in this study, only 20 tools (6 developed in high-income countries and 14 developed in LMICs) were identified. They varied significantly in their psychometric performance and feasibility. None of the tools fully met all the criteria established by the experts. Four tools (DSS, GMCD, MDAT, and Ten Questions Questionnaire) had good psychometric properties (over 80%). However, only three of these (GMCD, MDAT, and Ten Questions Questionnaire) met most items of feasibility listed by the experts. An important limitation of this study is that authors not always provide in their publications the information required for a fuller assessment of the feasibility of applying the tools.

**Table 1.** Mean, median, and range of experts' responses to items regarding feasibility in LMIC

Characteristics	Mean	Median	Range
Results understood by health workers	4.64	5	1-5
Reliable	4.61	5	2-5
Valid	4.59	5	2-5
Acceptable to caregivers	4.59	5	1-5
Provides information that is relevant to primary care providers	4.52	5	2-5
Information that can be used for referrals of early intervention	4.50	5	2-5
Information that is useful for anticipatory guidance	4.45	5	1-5
Results understood by caregivers	4.36	5	1-5
Staff members have the expertise to answer questions	4.23	5	1-5
Access to application	4.18	4.5	1-5
Training involved	4.14	4	2-5
How long it takes to administer the tool	4.12	4	3-5
Cover multiple areas of child development	4.07	4	2-5
Cost of the tool	4.02	4	1-5
Minimal adaptation needed	3.50	3	1-5
Educational level of staff members	3.45	3	2-5
How many staff members to administer the tool	3.40	3	2-5
Local norms available	3.24	3	1-5
Space	3.02	3	1-5

**Table 2.** Evaluation of tools according to feasibility criteria

Characteristics	GMCD	MDAT	Ten Questions Questionnaire
Costs of the instrument	Not mentioned	1	1
Access to application	Not mentioned	1	Not mentioned
Training involved	1	1	1
Results are useful to guide action	1	1	0
Time for application	1	1	Short
Results are understood by caregivers and workers	1	Not formally, but yes	1

Assessing children at PHC for developmental disabilities holds the promise of identifying and referring for more specialized care to children who can benefit from existing interventions. The inclusion of such an assessment at PHC would respond to concerns held by parents and also by countries that want to take action to improve outcomes in such children.

When assessing children for developmental disabilities, it is important to consider the skills and knowledge required to administer the tool as well as the time required for its administration. High-skilled testers are expensive to train and often in very limited supply for deployment at PHC. Also, the demand for services requires that the assessment be completed within the time of the consultation—that usually is shorter than 20 minutes. In addition, the assessment should not replace counselling required for clear communication of results and promotion of stimulating initiatives by the family and avoiding stigmatization of children identified at risk of developmental delay.

The tools developed in western countries described in this manuscript have strong psychometric performance but, in addition to their adaptation needs, their costs, training requirements, and time for application make these less suitable for use at PHC in LMICs.

Examining the feasibility of tools, nonetheless, is only relevant when the good technical performance of the tool has already been demonstrated. Accordingly, psychometric performance of the tools was scored as highly important by the consulted experts. Another highly-valued characteristic was the tool's specificity as a false positive indication of developmental disorder that can have significant undesirable consequences for the child and the family.

It was noticeable that the experts did not rate highly the need for tool adaptation and for availability of local norms. This may be related (as indicated by one of the experts) to the expectation that internationally-applicable norms would be available and that adaptation of the selected tools would be simple.

Out of the 14 tools developed in LMICs that we reviewed, none fully met the feasibility criteria proposed by the experts. Only four tools (DSS, GMCD, MDAT, and Ten Questions Questionnaire) were found to have adequate overall psychometric properties defined as sensitivity and specificity equal to or greater than 80%. These tools tended to be of greater feasibility than western tools in terms of costs, access, demands on health workers’ time, and training requirements. These also appeared to suit the characteristics of service delivery in PHC settings in LMICs. Nonetheless, a few improvements would make these tools more suitable for implementation. In the case of the GMCD, it has been shown to be feasible in the Turkish context. Cross-cultural validation is under process and will provide important information to guide the decision on whether to recommend its use to other settings. Also, information from new studies on whether the results are understandable by caregivers and PHC workers and if they are used in guiding action would improve the confidence in its feasibility. The MDAT is a comprehensive tool with good psychometric properties. As a screening tool, however, it has its feasibility diminished because it takes 35 minutes to apply. The creation of a shorter version might be a good strategy to allow its use in PHC in LMIC settings. The Ten Questions Questionnaire is short and simple to be used and has already been culturally validated. However, this is limited for application among 0 to 2 year(s) old children only. It has good sensitivity to pick up serious cognitive, motor and seizure deficits but lower sensitivity for vision and hearing deficits warrant inclusion of separate hearing and vision screens.

### Conclusions

The assessment of child development when linked to interventions to manage children with disabilities will help increase the proportion of children that fulfill their developmental potential. However, well-performing tools to identify children requiring intervention are needed. As the correct identification of children with developmental disability in their first years of life may help take action to reverse or reduce the impact of impairments, an incorrect assessment can cause significant harm to children and their families. Investment in research and development to improve the performance of existing tools and their feasibility for application in primary healthcare settings is a priority if the benefits of interventions to address disabilities are to be extended to more children in LMICs.

## Annex. Terms used for search on databases

(((((“Child Development”[Mesh] OR “infant development”[TW] OR “child development*”[TW] OR “child neuro development”[TW]))

and

(“Assessment”[TW] OR “tool”[TW] OR “scale”[TW] OR “Instrument”[TW] OR “questionnaire”[TW] OR “checklist”[TW] OR “monitoring”[TW] OR “Technology Assessment, Biomedical”[Mesh] OR “Questionnaires”[Mesh] OR “screening”[TW]))

or

(((((“Child”[Mesh] OR “Maternal-Child Health Centers”[Mesh] OR “Child Health Services”[Mesh] OR “Child Care”[Mesh] OR “Child, Hospitalized”[Mesh] OR “Child Development”[Mesh] OR “Child Behavior”[Mesh] OR “children”[TIAB] OR “newborn*”[TIAB] OR “childhood”[TIAB] OR “baby”[TIAB] OR “babies”[TIAB] OR “toddler”[TIAB] OR “toddlers”[TIAB] OR “infants”[TIAB] OR “infant”[TIAB] OR “infantile”[TIAB] OR “young patient”[TIAB] OR “young patients”[TIAB] OR “Hospitals, Pediatric”[Mesh] OR “Pediatrics”[Mesh] OR “Pediatricians”[TIAB] OR “paediatricians”[TIAB] OR “Pediatrician”[TIAB] OR “paediatrician”[TIAB] OR “Pediatrics”[TIAB] OR “paediatrics”[TIAB] OR “Pediatric”[TIAB] OR “paediatric”[TIAB]))

and

(“Developmental Disabilities”[Mesh] OR “developmental delay*”[TW] OR “developmental disabilit*”[TW] OR “developmental difficult*”[TW] OR “neurodevelopmental disorder*”[TW] OR “neurodevelopmental disability*”[TW] OR “developmental milestone*”[TW])))

and

(“Assessment”[TW] OR “tool”[TW] OR “scale”[TW] OR “Instrument”[TW] OR “questionnaire”[TW] OR “checklist”[TW] OR “monitoring”[TW] OR “Technology Assessment, Biomedical”[Mesh] OR “Questionnaires”[Mesh] OR “screening”[TW])))
